# Books and Authors

**Published:** 1906-07

**Authors:** 


### BOOKS AND AUTHORS.

**A Textbook of Obstetrics.** By **Adam H. Wright,** Professor of Obstetrics, University of Toronto; Obstetrician and Gynecologist to the General Hospital, Toronto, Canada. With two hundred and twenty-four illustrations in the text. Octavo, 591 pages. New York and London: D. Appleton and Company. 1905. (Price: Cloth, $4.50).

Textbooks of obstetrics are not scarce nor are they weak at this present time. A number of excellent works in this field have appeared within the last ten years, some of which have been of a pretentious character. Professor Wright, whose experience as a teacher of obstetrics extends over a period of thirty years, has presented an exceedingly modest treatise; nevertheless, it takes rank with the practical works which are and ever will be most popular with undergraduate students, and with practitioners who attend labor cases.

The author makes two grand divisions of his subject,—first, physiological obstetrics; and, second, pathological and operative obstetrics. The nine chapters in the first section consider in their order anatomy, physiology, the embryo and fetus, pregnancy, physiology of labor, management of normal labor (two chapters), the puerperal state and, finally, face presentations, breech presentations, and multiple pregnancies. This is a systematic arrangement of sub-topics, and each is given space and consideration according to its importance. Very properly, we think, anatomy and physiology are given limited space, being summarised in fact, while clinical midwifery is considered in greater detail. For example, normal labor is handled with minute exactitude, and this remark may apply with equal cogency to the consideration of pathological and operative obstetrics in the next grand division.

Passing on to the second section we find the topics allotted to it dealt with in seventeen chapters, taking up about two-thirds of the volume. They are the diseases of pregnancy, intercurrent diseases of pregnancy, diseases of pregnancy and the puerperium, extrauterine pregnancy, the hemorrhages, abortion or miscarriage, prolonged and precipitate labor, malpositions and abnormal conditions of the fetus, abnormal conditions of the uterus, etc., emotional elements in the puerperal period and puerperal insanity, Listerism and obstetrics, puerperal infection, deformities of the bony pelvis and injuries to the child during delivery, major and minor obstetrical operations,—the last three chapters being devoted to the latter two topics.

In regard to cardiac diseases Wright has given an interesting exposition. It has been thought by most obstetricians that heart lesions of any kind were inhibitive of pregnancy and even of marriage. Our author says that a woman even with valvular disease should be permitted to marry, with certain exceptions. When a heart lesion is compensated and no complication exists, then marriage need not be forbidden. On the other hand, if there is dyspnea, breathlessness, palpitation on exertion or hemoptysis, then matrimony should not be permitted. We have alluded to this important question for the purpose of drawing attention to it, and to point out the care Wright has taken to prepare a practical, clinical treatise, that should reflect the best modern thought on even some points that are rarely dealt with in similar works. It would be interesting to note many other features of this work wherein it excels, and especially wherein it differs from the usual treatise on obstetrics, but we recognise that even a review must be concise and that, after all, the book itself must be seen and examined to be appreciated.

We must not fail, however, to invite attention to the illustrations which, in the main, partake of the same practical character as the text. In particular is this the case with those delineating the repair of the pelvic floor and perineum. They are drawn from life and, therefore, illustrate actual cases. The entire work is one that cannot fail to please the student of obstetrics, whether undergraduate or more advanced practitioner, and constitutes a distinct addition to the literature of the subject.

**International Clinics.** A Quarterly of Illustrated Clinical Lectures and especially prepared articles on Treatment, Medicine, Surgery, Neurology, Pediatrics, Obstetrics, Gynecology, Orthopedics, Pathology, Dermatology, Ophthalmology, Otology, Rhinology, Laryngology, Hygiene and other topics of interest to students and practitioners. By leading members of the medical profession throughout the world. Edited by **A. O. J. Kelly,** A.M., M.D., Philadelphia. Volume I. Sixteenth series. 1906. Philadelphia and London: J. B. Lippincott Co. (Cloth, $2.00).

Under the head of treatment this volume contains the following articles: Medical treatment of exopthalmic goiter, by James Tyson; treatment of gastroptosis, by Albert Philip Francine: coughing and its relation to treatment, by James M. French; dechloridation treatment of diseases of the heart, by Ernest Barie: indication for, and methods of, performing venesection, by John W. Wainwright. The articles under the title of Medicine are, diagnosis and treatment of membranous tonsillitis, by Lewis S. Somers; position and size of the heart in advanced mitral stenosis, by M. Howard Fussell; origin and preventive treatment of oxalic acid deposits in the urine, by G. Klemperer; death and blindness as a result of poisoning by wood alcohol and its various preparations, by Casey A. Wood.

On Surgery the titles are, A method of lengthening the tendo Achilles and other tendons, by Russell A. Hibbs; gonorrheal synovitis,—carcinoma of the pyloric end of the stomach, by Nicholas Senn; some effects of quiet renal calculus, by William Henry Battle; treatment of carcinoma of the tongue, by J. L. Faure; importance of a study of the pulse in surgical diseases, etc., by Charles Greene Cumston. The articles on obstetrics and gynecology are, causation and treatment of eclampsia with special reference to rapid delivery, by Joseph B. DeLee; chorio-epthe-liomia malignum in a pregnant uterus, by Ernest Boyen Young. On pathology, there is one article—a contribution to the study of eosinophilia, by Charles E. Simon.

The progress of medicine during 1905 in relation to treatment is dealt with by A. A. Stevens; in regard to medicine, by David L. Edsall and Verner Nisbet; and in surgery, by Joseph C. Bloodgood. It will be observed by this list of titles and authors that this is one of the best of these volumes lately published. Dr. DeLee's article on rapid delivery of the fetus in eclampsia is worth the price of the series for a year. Several of the other contributions also possess exceptional merit.

---

**Food and Principles of Dietetics**. By **Robert Hutchison**, M.D., Edinburg, F.R.C.P.; Assistant Physician to the London Hospital, and to the Hospital for Sick Children, Great Ormond Street, etc. With plates and diagrams. Revised second edition. Octavo, 582 pages. New York: William Wood and Company. 1906. (Price: cloth, $3.00 net).

It is, at this writing, five years and a half since the first edition of this work was presented to the profession, and many changes in methods and practice have occurred during that time. Hutchison is well known to the medical world as a teacher and writer, and as a man who would not rest content to have his book fall behind in the march of progress. He has, therefore, brought it up to the present date by revising this edition in a careful manner.

The treatise embodies the author's lectures at the London hospital, but he has added thereto much material not included in the lectures as delivered to the students. Indeed, as now published it is not only suited to the requirements of students and practitioners of medicine, but it is adapted to the needs of anyone who may desire to acquire an intelligent acquaintance with many problems of nutrition or to obtain proper knowledge of food values.

The foods derived from milk, their digestibility and effect on nutrition, are presented most completely and interestingly. In regard to cheese-making, Hutchinson says at present it is a rule-of-thumb process; by and by it will become a science. He says the cheesemaker of the next century will have a laboratory attached to his factory in which pure cultures of each variety of cheese will be nursed, and the bacteria responsible for every flavor will be produced, so that all varieties of cheese will be made under one roof. We have referred to this one topic to indicate the thoroughness of the author and the general value of his book.

It seems to us this work should be purchased by every public library and its reading should be encouraged by every teacher. It should form the basis of teaching the physiology of life and the preservation of health in our public schools. Physicians everywhere should encourage this view, and should begin by becoming familiar with it themselves. Its price is such as to place it within the reach of even the slender purse, while its quality and make-up are beyond criticism.

---

**Diseases of Infancy and Childhood.** By **L. Emmett Holt,** M.D., Sc.D. LL.D., Professor of Diseases of Children in the College of Physicians of Columbia University, New York. With 241 illustrations, including eight colored plates. Third edition revised and enlarged. Octavo, pp. 1174. New York and London: D. Appleton. 1906. (Price, $6.00).

Holt has been accepted for a considerable period of time as authority on questions relating to diseases of children, and is, perhaps, as often quoted on this topic as any author of the present day. His work is generally accepted as a textbook in the schools and is, likewise, a safe guide for the general practitioner.

The improvements made of late and the advances constantly making in the field of pediatrics, require frequent editions of such a work in order to keep it abreast of the science. Holt has industriously and conscientiously revised his treatise until very little remains to be done that could improve it or make it a better exponent of pediatric knowledge. It is a book for student and practitioner rather than for specialist, but the latter cannot afford to be without it.

Holt has reason to feel pride in the fact that more than fifty thousand copies of the work have been printed since its first issue —a showing not only remarkable in itself but in the further fact that this record has never been equaled by any other work on pediatrics. Among other improvements in this edition we must not forget the illustrations, which are of decided merit. New pictures have been introduced, and old ones have exchanged places with better and sharper cuts, until nothing more seems to be desired in this direction—indeed, the book as a whole has attained an excellence unsurpassed by any American or foreign treatise on diseases of infants and children.

---

**A Syllabus of Materia Medica.** By **Warren Coleman,** M.D., Professor of Clinical Medicine in Cornell University Medical College. Second edition. New York: William Wood & Co. 1905. (Price, $1.00).

This little volume serves as an excellent reminder of the essentials of the most important drugs and their uses. Two new sections have been added to this edition, one on minor toxic actions, and the other on toxicology. The work has also been subjected to careful revision. It is helpful to the student in pursuing the study of materia medica and especially so to the practitioner in refreshing his memory when he may not need to consult larger works.

---

**Memoranda on Poisons.** By **Thomas Hawkes Tanner,** M.D., F.L.S. Tenth revised edition by **Henry Leffmann,** A.M., M.D., Professor of Chemistry in the Woman's Medical College of Pennsylvania. Philadelphia: P. Blakiston's Son & Co. 1905. (Price, $0.75).

The usefulness of this little volume is apparent from its title and the ten editions through which it has passed. Every physician needs such a reference booklet, because none can carry the details of management in cases of poisoning from all the toxic materials that are liable to be taken by accident or design. This edition has been revised to meet the advances and changes and is as complete as the limited nature of the work will permit.

---

**Nasal Sinus Surgery with Operations on Nose and Throat.** By **Beaman Douglass,** M.D., Professor of Diseases of the Nose and Throat in the New York Post-Graduate Medical School and Hospital. Illustrated with 68 full-page Half-tone and Colored Plates, including nearly 100 Figures. Royal Octavo, 256 pages. Bound in Extra Cloth. F. A. Davis Company, Publishers, 1914-16 Cherry St., Philadelphia, Pa. (Price: $2.50 net).

The importance of the topics discussed in this book is of a growing nature, and nowhere has the surgery of the sinuses been set forth clearer or with more scientific emphasis than in the volume before us. A general anatomical review of the nose begins the treatise, which is distinguished for its clearness and for the thoroughness of description given, supported also by the best illustrations we have seen.

The frontal sinus forms the subject of the second chapter, which is a concise review of its anatomy and surgery. The rule for finding the sinus is excellent and the diagram makes it perfect. The third chapter deals with the ethmoidal sinus and is equally as meritorious as the preceding, the descriptive anatomy and the surgical procedures being of the first order, all beautifully illustrated. The maxillary sinus is next considered, the operations on the antrum being of special interest.

The fifth chapter is concerned with the sphenoidal sinus and presents in graphic detail the anatomy and surgery of this cavity and, like the others, is superbly illustrated. The next following chapters, down to the tenth, deal with the nose and throat surgery of every day practice, such as nasal deformities, deflections of the septum, turbinectomy, tonsillotomy, and the like; while the tenth chapter is devoted to laryngotomy and tracheotomy.

Douglass has written a work that is unlike any other,—one that limits itself to the title which he has given it,—and it may be said to be the most practical dissertation of surgical laryngology that has appeared in English. Moreover, it is admirably put together, beautifully illustrated, and handsomely printed.

